# Mitochondrial dysfunction as a possible trigger of neuroinflammation at post-traumatic stress disorder (PTSD)

**DOI:** 10.3389/fphys.2023.1222826

**Published:** 2023-10-24

**Authors:** Tetiana R. Dmytriv, Sviatoslav A. Tsiumpala, Halyna M. Semchyshyn, Kenneth B. Storey, Volodymyr I. Lushchak

**Affiliations:** ^1^ Department of Biochemistry and Biotechnology, Vasyl Stefanyk Precarpathian National University, Ivano-Frankivsk, Ukraine; ^2^ Department of Biology, Institute of Biochemistry, Carleton University, Ottawa, ON, Canada; ^3^ Research and Development University, Ivano-Frankivsk, Ukraine

**Keywords:** post-traumatic stress disorder (PTSD), mitochondria, inflammation, hypothalamic-pituitary-adrenal axis (HPA), steroidogenesis, monoamine oxidase A

## Abstract

Post-traumatic stress disorder (PTSD) is a neuropsychiatric disorder that occurs in approximately 15% of people as a result of some traumatic events. The main symptoms are re-experiencing and avoidance of everything related to this event and hyperarousal. The main component of the pathophysiology of PTSD is an imbalance in the functioning of the hypothalamic-pituitary-adrenal axis (HPA) and development of neuroinflammation. In parallel with this, mitochondrial dysfunction is observed, as in many other diseases. In this review, we focus on the question how mitochondria may be involved in the development of neuroinflammation and its maintaining at PTSD. First, we describe the differences in the operation of the neuro-endocrine system during stress versus PTSD. We then show changes in the activity/expression of mitochondrial proteins in PTSD and how they can affect the levels of hormones involved in PTSD development, as well as how mitochondrial damage/pathogen-associated molecule patterns (DAMPs/PAMPs) trigger development of inflammation. In addition, we examine the possibility of treating PTSD-related inflammation using mitochondria as a target.

## 1 Introduction

Post-traumatic stress disorder (PTSD) is a prolonged reaction to the extremely stressful events. Traumatic events like combat exposure, sexual assault, or life-threatening illnesses (among others) can cause stress-related mental disorders which can lead to PTSD development. People with PTSD may have recurring and intrusive memories or nightmares of the traumatic event, and may also experience anxiety, depression, hyperarousal, and avoidance behaviors. These symptoms can be severe and interfere with daily activities (Arnberg et al., 2013). It worth to note, that other pathophysiological conditions can have similarities with PTSD. For example, in case of obesity, adipose tissue releases proinflammatory molecules called adipokines which can travel to the brain and contribute to neuroinflammation (Salas-Venegas et al., 2022). Also, aging is associated with chronic low-grade neuroinflammation, that can be referred to as “inflammaging” (Andronie-Cioara et al., 2023). Despite some PTSD features are fundamentally different from ones at obesity and aging, many of them are common between these conditions.

Due to the importance of this topic, scientists worldwide have offered several hypotheses that explain the origin and development of PTSD. For example, the hypothalamic-pituitary-adrenal axis (HPA) dysregulation hypothesis postulates that impairment of the HPA axis is the main trigger for PTSD development. This is the neuroendocrine pathway that takes place in every stress response via regulation of secretion of glucocorticoids (Dunlop and Wong, 2019). The epigenetic hypothesis suggests that PTSD is characterized by alterations in gene expression resulting from exposure to traumatic stress followed by the long-term influence of the environment on gene expression (Howie et al., 2019). Indeed, there is increasing evidence from PTSD patients regarding the role of epigenetic changes regulation of individual gene expression and pathways in the pathogenesis of PTSD (Howie et al., 2019; Cao et al., 2022). It is important to note that these hypotheses are not mutually exclusive, and there is likely significant overlap between them as exemplified by epigenetic regulation of the expression of genes related to the HPA axis.

A growing body of research shows links between PTSD and mitochondrial dysfunction (Bersani et al., 2016; Mellon et al., 2019; Hummel et al., 2023). In particular, it has been established that individuals with PTSD have impaired mitochondrial function and this can trigger development of oxidative stress and inflammation (Lushchak et al., 2023). In turn, disregulation of mitochondrial function affects the levels of neurotransmitters, including those involved in mood regulation such as serotonin and dopamine. This contributes to symptoms such as depression and anxiety, which are commonly experienced by individuals with PTSD (Zhang et al., 2006; Bersani et al., 2020). Furthermore, mitochondrial dysfunction may be associated with other factors related to the development of PTSD, such as a genetic predisposition. This leads to a vicious cycle where mitochondria accelerate PTSD symptoms, and *vice versa*.

Importantly, mitochondrial dysfunction in neuronal tissue is linked with neuroinflammation. The latter is an innate reaction of the body that is a protective mechanism against infection or damage, thereby ensuring homeostasis in the central nervous system while, at the same time, being one of the key mechanisms in the development of neurodegenerative diseases (DiSabato et al., 2016). Imbalance in the operation of the HPA axis may trigger a pro-inflammatory response leading to chronic inflammation and damage to brain cells that may develop into PTSD. Normally, HPA is responsible for the secretion of glucocorticoids during stress, in particular, cortisol. This leads to inhibition of lymphocyte proliferation and as a result—a decrease in the levels of pro-inflammatory cytokines such as IL-6, IL-12, and tumor necrosis factor α (TNF-α). Decrease in cortisol levels is one of PTSD signs. In this regard, an imbalance in the work of HPA, on the contrary, leads to an increase in the level of the mentioned pro-inflammatory cytokines and development of neuroinflammation. Both, neuroinflammation and mitochondrial dysfunction, are inherent to PTSD (Lushchak et al., 2023).

This review aims to disclose the role of mitochondria in the regulation of immune function and neuroinflammation with a particular interest in mitochondrial dysfunction as a contributor to the development of chronic neuroinflammation associated with PTSD.

### 1.1 Perturbations in neuroendocrine system under acute stress/PTSD and their possible role in development of neuroinflammation

The HPA axis acts as a central coordinator of neuroendocrine changes in response to stressors. It consists of endocrine hypothalamic components, including the anterior part of the pituitary and adrenal glands. During a stress event, the neurons of the paraventricular nucleus of the hypothalamus secrete corticotropin-releasing hormone (CRH), that stimulates the secretion of adrenocorticotropic hormone (ACTH) from the anterior lobe of the pituitary gland (Leistner and Menke, 2020). ACTH then travels through the bloodstream to the adrenal glands inducing their release of cortisol, epinephrine, and norepinephrine (Hadad et al., 2020; Leistner and Menke, 2020). Cortisol and epinephrine modulate metabolic processes and immune activity in the brain, mobilizing energy resources to provide physiological and behavioral response to stress, commonly called “the fight or flight reaction”. It is important to note that cortisol levels are regulated by a negative feedback loop that inhibits further cortisol production in response to the stress event (Keller-Wood, 2015). Epinephrine and norepinephrine cause the heart to beat faster and harder, which increases blood flow and oxygen delivery to muscle (Bremner, 2006; Wong et al., 2012). Thus, the organism uses available energy to meet the challenging event.

It is worth noting the contribution of the orexinergic system for functioning of HPA. During stressful situations, orexin release can help maintain alertness and cognitive function which can be beneficial for responding to the stressor effectively (Milbank and López, 2019). At the same time, chronic dysregulation of the orexinergic system can contribute to sleep disorders, such as insomnia (Brown et al., 2012).

**FIGURE 1:**
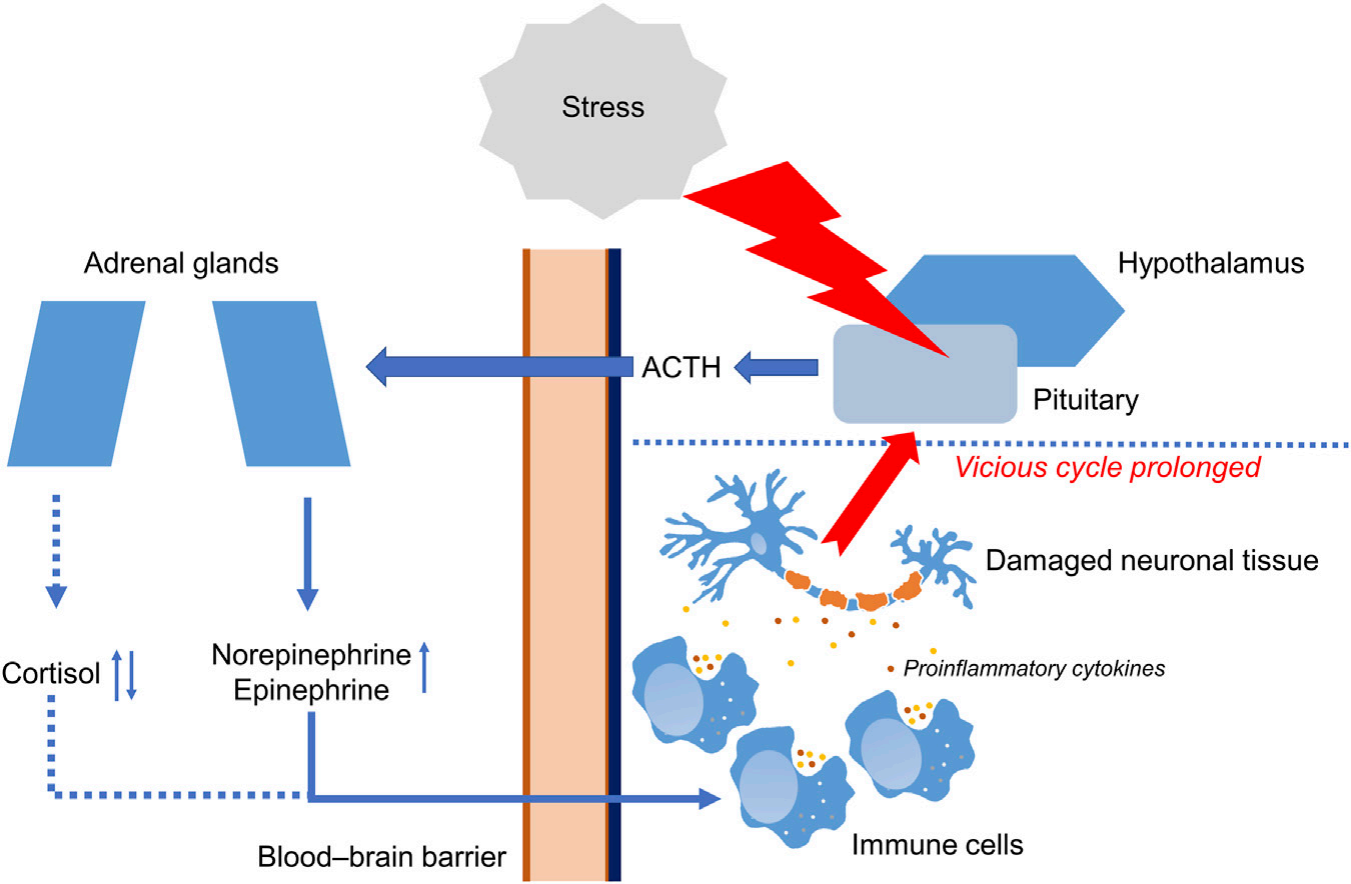
Functioning of the hypothalamic-pituitary-adrenal axis in PTSD. Stressful event causes molecular and cellular changes in hypothalamic–pituitary–adrenal axis (HPA). In response to stress, HPA leads to secretion of adrenocorticotropic hormone (ACTH) crossing blood–brain barrier (BBB) and stimulating adrenal glands. Then, adrenal glands increase production of cortisol, norepinephrine, and epinephrine. Changes in the work of HPA lead to the production of pro-inflammatory cytokines such as IL-1β and IL-6 by immune cells. Prolonged inflammation may lead to damaging of neuronal tissue. ↑ Increased during PTSD. ↓↑ Decreased or increased during PTSD.

Acute stress and PTSD can both affect the HPA axis but in different manners. Acute stress is a normal response to a perceived threat or challenge, and is characterized by a rapid activation of the HPA axis. By contrast, PTSD dysregulates the HPA axis (Mouthaan et al., 2014; Morris et al., 2016; Dunlop and Wong, 2019). Figure 1 shows the functioning of the HPA axis in PTSD.

Disruption of the HPA axis can lead to the excessive production of proinflammatory cytokines IL-1β, IL-6, IL-12, and TNF-α (Lushchak et al., 2023) triggering development of neuroinflammation. Inflammation encompasses various physiological and pathological processes that are orchestrated by mediators which form intricate regulatory networks (DiSabato et al., 2016). Neuroinflammation arises as a result of overstimulation of the adaptive immune system in the brain. The agents that drive a neuroinflammatory response are immune cells (microglia) in the brain, that can be activated by inflammatory mediators, usually cytokines. For example, IL-6, IL-8, and TNF-α are all pro-inflammatory cytokines that can stimulate inflammation and promote the recruitment of immune cells to the action site (Na et al., 2014). IL-18 is another pro-inflammatory cytokine that is involved in the activation of the immune response and regulation of inflammatory processes (Amarachi Ihim et al., 2022). Prolonged cytokine release leading to chronic inflammation causes tissue damage and contributes to the pathogenesis of many chronic diseases (Na et al., 2014; DiSabato et al., 2016). Inflammation also leads to an excessive production of reactive oxygen species (ROS), that may cause exacerbation of inflammation. Furthermore, chronic stress enhances the development of neurodegenerative diseases (Garaschuk et al., 2018).

As mentioned above, cortisol is one of the key stress hormones with anti-inflammatory action. Its low level is supposed to play a crucial role in PTSD development. Administration of cortisol in order to normalize its level may prevent development of PTSD-like symptoms (Morris et al., 2016). Cortisol can bind to specific receptors that are present on immune cells and dampen the immune response (Castro-Vale et al., 2016; Morris et al., 2016). Furthermore, cortisol can induce the expression of anti-inflammatory factors, such as lipocortin-1, that can inhibit the activity of enzymes involved in the production of inflammatory mediators such as prostaglandins and leukotrienes (Buckingham and Flower, 1997; Rhen and Cidlowski, 2005).

### 1.2 The role of mitochondria in the work of the neuroendocrine system and development of neuroinflammation

Operation of the neuroendocrine system and, in particular, the HPA axis depends to some extent on mitochondria. First of all, this is due to the fact that mitochondria in the cortex of adrenal glands take part in the biosynthesis of all steroid hormones, including glucocorticoids (Picard et al., 2018). Their main product in humans is cortisol, whereas in rodents it is corticosterone. An increase in the concentration of glucocorticoids is a classic response to stressful challenges that provides adaptive mobilization of energy resources and anti-inflammatory effects (Whirledge and Cidlowski, 2010). However, it is now known that both higher (Tucker et al., 2004; Friedman et al., 2007; Ogłodek, 2022) and lower (Glover and Poland, 2002; Bremner et al., 2003; Olff et al., 2007; Golier et al., 2014) cortisol levels in urine, saliva or blood plasma can be observed in PTSD or, in some case, show no change (Otte et al., 2007). In addition, mitochondria are involved in the catabolism of such catecholamines as serotonin, dopamine, epinephrine, and norepinephrine (Picard et al., 2018). For example, serotonin levels can be decreased (Seidemann et al., 2021; Ogłodek, 2022) and norepinephrine increased (Geracioti et al., 2001; Friedman et al., 2007; Pan et al., 2018) in blood serum, urine, and cerebrospinal fluid during PTSD and potentially the total levels of these hormones may depend not only on the rate of biosynthesis but also on mitochondrial catabolism.

We hypothesize that an imbalance in operation of the neuroendocrine system during PTSD could be related to changes in the activity and/or expression of mitochondrial proteins involved in the biosynthesis of steroid hormones and catabolism of catecholamines. Because the mitochondria participate in the biosynthesis and catabolism of the above-mentioned hormones, that are considered anti-inflammatory (cortisol, norepinephrine) or pro-inflammatory (serotonin) (Hannibal and Bishop, 2014; O’neill and Harkin, 2018; Kanova and Kohout, 2021), these hormones can influence/induce neuroinflammation in PTSD. In addition, it has long been known that they can contribute directly to the development of inflammation by various mechanisms which will be discussed further.

#### 1.2.1 Mitochondria as a regulator of the biosynthesis and catabolism of hormones are involved in the development of PTSD symptoms and related inflammation

**FIGURE 2:**
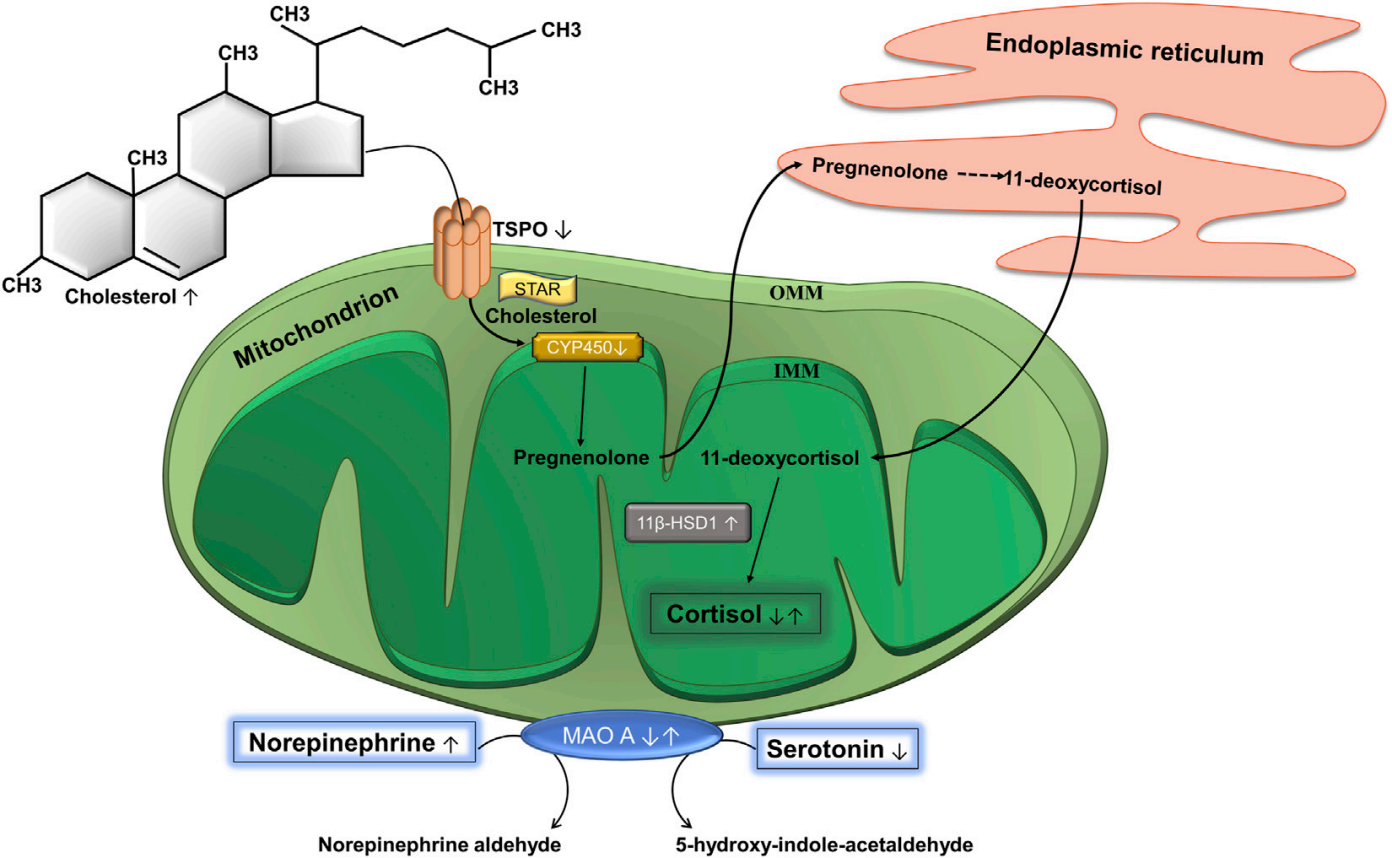
Biosynthesis of cortisol and catabolism of catecholamines. Cholesterol is the precursor of cortisol and the translocator protein (TSPO) complex is a site of entry of cholesterol into mitochondria. It is located on the outer mitochondrial membrane (OMM). Steroidogenic acute regulatory protein (STAR) moves cholesterol from OMM to the inner mitochondrial membrane (IMM), where cytochrome P450 (CYP450) is located. CYP450 converts cholesterol into pregnenolone, which is then transported to the endoplasmic reticulum, where it is converted to 11-deoxycortisol through a series of reactions. The latter gets back into the mitochondria, where the enzyme 11β-hydroxysteroid dehydrogenase 1 (11β-HSD1) catalyzes the conversion of 11-deoxycortisol into cortisol. Mitochondria are also involved in the catabolism of catecholamines. The OMM contains the enzyme monoamine oxidase A (МАО А), which catalyzes the oxidative deamination of catecholamines, in particular, serotonin and norepinephrine to the corresponding aldehydes. ↓ Decreased at PTSD. ↑ Increased at PTSD. ↓↑ Decreased or increased at PTSD.

The biosynthesis of glucocorticoids and, in particular, cortisol is a multistage process that takes place with the participation of two cell organelles: mitochondria and endoplasmic reticulum (Figure 2). The first and key stage of steroidogenesis is transport of the precursor of all steroid hormones (cholesterol) to the inner mitochondrial membrane (IMM) (Rone et al., 2009). This takes place in two consecutive steps. In the first step, translocator protein (TSPO), a transmembrane protein located on the outer mitochondrial membrane (OMM) and possessing a cholesterol-binding domain, transports cholesterol through the OMM (Biswas et al., 2018). TSPO is expressed mainly in steroidogenic cells and is spatially located next to the mitochondrial pore (Papadopoulos et al., 2015). Then, in the second step cholesterol is transported from the OMM to the IMM. This process is carried out by steroidogenic acute regulatory protein (StAR) that is a kind of contact site between the OMM and IMM and is a rate-limiting stage of steroidogenesis (Miller, 2007). Next, cholesterol is processed by the first steroidogenic mitochondrial enzyme—cytochrome P450 (CYP450). This enzyme splits the side chain of cholesterol that is located on the matrix side of the IMM and catalyzes the conversion of cholesterol to pregnenolone. This process of catalysis takes place in three consecutive reactions: 20-hydroxylation, 22-hydroxylation, and breaking of the 20–22 carbon bond. Each reaction requires a pair of electrons, which CYP450 receives from NADPH. First, NADPH is bound by ferredoxin reductase on the IMM, that interacts with the protein ferredoxin and transfers a pair of electrons to it. Ferredoxin then mediates the transfer of this pair of electrons to CYP11A1. Thus, the process of converting cholesterol into pregnenolone depends entirely on the supply of electrons by the NADPH-ferredoxin reductase-ferredoxin system (Miller, 2005). Pregnenolone is the first steroid synthesized in the process of steroidogenesis (Bassi et al., 2021). It is believed that it freely leaves the mitochondria without the participation of any transport protein and enters the endoplasmic reticulum, that is spatially located next to mitochondria. Further stages of steroidogenesis take place in the mitochondria with the participation of a large family of homologous enzymes (∼57 in humans)—the cytochrome P450 hydroxylases. As a result of several reactions, that we will not dwell on, 11-deoxycortisol is formed from pregnenolone, and 11-deoxycortisol is transferred back into the mitochondria, where the final stages of steroidogenesis occur (Acconcia and Marino, 2016). Here, two isozymes play key roles, 11β-hydroxysteroid dehydrogenase (11β-HSD) 1 and 2. They catalyze the interconversion of active and inactive forms of glucocorticoids: 11β-HSD1 converts the inactive form 11-deoxycortisone into active cortisol, enhancing the cellular response, and 11β-HSD2 catalyzes the reverse reaction, inactivation of cortisol to 11-deoxycortisone (Chapman et al., 2013).

It is possible that changes in cortisol concentration associated with PTSD may be related to steroidogenesis inside mitochondria because the intracellular cholesterol pool of steroidogenic cells regulates the biosynthesis of steroid hormones (Bassi et al., 2022). A systematic meta-analysis of lipid signatures in PTSD showed that total cholesterol level was increased (Bharti et al., 2022). In general, an increase in cholesterol level could indicate that CYP450 has a sufficient amount of available substrate to initiate the first reaction of steroidogenesis, since its activity depends on the availability of substrate (Jefcoate, 2002). However, the availability of substrate in this case will also depend on whether it is efficiently transported into the mitochondria. For example, PTSD patients have been found to have lower availability/expression of TSPO in the brain as compared to controls (Bhatt et al., 2020). Interestingly, the use of TSPO agonists can be used to treat PTSD and effectively relieve symptoms. A study of the mechanisms showed that this may be related to an increase in glucocorticoid levels (Shang et al., 2020).

Another important protein involved in cholesterol transport is STAR, however, unfortunately, there is no any data available on its changes in PTSD, although this stage limits the rate of steroidogenesis (Miller, 2007). Also, in one of study, about 1,000 differentially expressed genes were identified in the hippocampus of mice that exhibited PTSD-like symptoms as a result of previous exposure to aggressor mice. Among them, the downregulation of the CYP1A2 gene expression was found which could potentially be one of the key mechanisms associated with PTSD (Bian et al., 2019).

The enzymes, 11β-HSD1 and 11β-HSD2, may also be related to this disorder. In PTSD, significantly higher levels of prefrontal-limbic 11β-HSD1 were found but this did not correlate with the level of peripheral cortisol in plasma (Bhatt et al., 2021). However, it was shown that 11β-HSD1, by providing high levels of glucocorticoids during stress, improved memory about fearful events and might contribute to the development of anxiety in PTSD. The use of a selective enzyme inhibitor, UE2316, led to a significant decrease in contextual fear memory induced by foot-shock conditioning in mice (Wheelan et al., 2015). 11β-HSD2 works in tandem with 11β-HSD1 and inactivates glucocorticoids. Interestingly, people with PTSD who survived the Holocaust, as well as their descendants, were found to have lower cortisol levels. 11β-HSD2 activity was highest in offspring whose mothers were children during the Holocaust, with progressively lower activity in offspring whose mothers were teenagers and adults. However, analysis showed that this increase in activity was only observed in offspring whose mothers were without PTSD, and PTSD did not affect 11β-HSD2 activity (Bierer et al., 2014). Holocaust survivors also possessed lower 11β-HSD2 activity, which was more related to age than to PTSD. The greatest decline was associated with an earlier age of exposure to the Holocaust and less severe PTSD symptoms (Yehuda et al., 2009).

Considering all the facts mentioned above, our hypothesis is as follows. Despite the elevated level of cholesterol (Bharti et al., 2022), it can be poorly transported through the OMM because of an insufficiency of TSPO (Bhatt et al., 2020). As a result, only a small part of the precursor of steroid hormones enters the mitochondria. The first reaction of steroidogenesis is limited by a downregulation of CYP1A2 (Bian et al., 2019), that could be a kind of adaptive response due to the low availability of the substrate. In addition, due to dysfunctions of the mitochondrial electron transport chain in PTSD (Su et al., 2008), the electrons necessary for the sequential three-step transformation of cholesterol into pregnenolone catalyzed by CYP1A2 (Miller, 2005) may not be supplied in sufficient amounts. However, unexpectedly, there may be an increased activity of the terminal enzyme of steroidogenesis 11β-HSD1taking place under stress conditions (Bhatt et al., 2021). Potentially, the reduction in cortisol levels could be associated with greater 11β-HSD2 activity but we found no evidence that it is altered in PTSD (Yehuda et al., 2009; Bierer et al., 2014). In general, it can be assumed that (a) the initial stages of steroidogenesis (transport through the OMM and the first reaction catalyzed by CYP1A2) limit the speed of the entire process, and (b) that the higher activity of 11β-HSD1 is a response to stress and cannot increase the concentration of cortisol if there is a lack of its precursor in mitochondria. In addition, increased 11β-HSD1 activity did not correlate with the level of peripheral cortisol (Bhatt et al., 2021). This hypothesis would explain the decrease in cortisol levels in PTSD.

Mitochondria are also involved in the catabolism of catecholamines (Figure 2). An integral membrane enzyme, monoamine oxidase (MAO), is located in the OMM. It is highly expressed in the liver, gastrointestinal tract, and neurons, where it provides detoxification of a number of endogenous and exogenous amines, and also catalyzes the oxidative deamination of various monoamines. There are two main MAO isoenzymes: MAO A and MAO B. The first shows greater affinity for hydroxylated substrates such as serotonin and norepinephrine, and the second for non-hydroxylated substrates such as benzylamine and beta-phenylethylamine (Finberg and Rabey, 2016).

Tseylikman and colleagues exposed rats to cat urine for 10 min every day for 10 days and their behavior and the activity of MAO A was measured (Tseylikman et al., 2017). The brains of rats susceptible to PTSD, possessed a lower MAO A activity than ones that were resilient to PTSD. In men, hypermethylation of the gene encoding MAO A in blood correlated with the severity of PTSD symptoms which could explain the increase in the level of norepinephrine and excessive activity of the noradrenergic system that ensures the appearance of the main symptoms of PTSD—repeated experiences and hyperarousal (Ziegler et al., 2018). However, a question arises here: if the activity of MAO A is decreased at PTSD, why does the level of norepinephrine increase, whereas serotonin decreases? First, there is information in the literature that pro-inflammatory cytokines can increase the activity of indolamine 2,3-dioxygenase (Christmas et al., 2011). This enzyme is the first and rate-limiting enzyme in the tryptophan degradation pathway. As a result, the amino acid tryptophan, which is a precursor of serotonin, is mainly metabolized to kynurenine under the action of indolamine 2,3-dioxygenase (Lee et al., 2022; Dell’Oste et al., 2023). Additionally, rats with PTSD had lower expression of tryptophan hydroxylase 1 in the hippocampus (Lee et al., 2021). This is the first and key enzyme of serotonin biosynthesis and catalyzes the hydroxylation of tryptophan (Kuhn and Hasegawa, 2020). As a result, increased indolamine 2,3-dioxygenase activity and reduced tryptophan hydroxylase 1 expression could result in conversion of tryptophan to kynurenine rather than to serotonin.

#### 1.2.2 Possible mechanism of induction of inflammation in PTSD: role of mitochondria

##### 1.2.2.1 Oxidative stress and mitochondrial DAMPs/PAMPs as key players of inflammation

Development of oxidative stress is an integral feature of the pathophysiology of PTSD (Lushchak et al., 2023; Reed and Case, 2023). Oxidative stress results from an imbalance between ROS formation and elimination leading to increased steady-state ROS levels and number of physiological consequences (Lushchak, 2014). The mitochondrial electron transport chain is the main source of ROS, which makes mitochondria the first target for many types of oxidative damages (Murphy, 2009) and that are also observed in many pathological conditions including PTSD (Garabadu et al., 2015; Kolesnikova et al., 2015). Using a rat model exposed to stress, it has been found that at PTSD, the level of lipid peroxides (one of the markers of oxidative stress) increased and the activity of the antioxidant enzymes, namely catalase and superoxide dismutase, decreased (Garabadu et al., 2015). Thus, oxidative stress increases in PTSD. In concert with mitochondrial dysfunctions, it is a coordinator and modulator of PTSD but at the same time it is involved in the progression of many other psychopathologies. Also, at PTSD like at stress in general, mitochondrial permeability increases (Lushchak et al., 2023). In particular, this can be associated with overexpression of the voltage dependent anion channel 1 (VDAC1), adenine nucleotide translocase (ANT1/2), and cyclophilin D (Cyp-D) in PTSD (Seo et al., 2019). VDAC1 forms the outer part of the mitochondrial permeability transition pore (mPTP). This channel ensures the entry of Ca^2+^, ATP, and other metabolites through the OMM into the cytosol. ANT1/2 was supposed to form the inner part of the mPTP, and CypD regulates mPTP closure (Jonas et al., 2017). In addition, VDAC1 is involved in outflux of superoxide anion radical from mitochondria to cytosol (Jezek and Hlavatá, 2005).

Due to increased membrane permeability and stress conditions mitochondrial-derived damage-associated molecular patterns (DAMPs) are released from mitochondria. They are endogenous molecules that are usually found inside mitochondria but under stressful or damage conditions can be released, triggering an intracellular signal transduction cascade, activating the expression of inflammatory mediators and development of inflammation. For example, DAMPs include mitochondrial DNA (mtDNA), ROS-modified cellular components, cytochrome *c*, and N-formylated peptides (Lin et al., 2022). So-called pathogen-associated molecule patterns (PAMPs) can also cause inflammation. They are usually exogenous ligands of microbial origin. It is broadly believed that mitochondria were formed as a result of the symbiosis of ancient bacteria with the ancestor of the eukaryotic cell, due to what they are suggested to be of bacterial origin (Meyer et al., 2018). Such hypothesis is argued by several arguments. For example, mtDNA, like bacteria, has a circular structure (Boguszewska et al., 2020). Mitochondrial proteins contain N-formylated amino acid residues that are mainly signs of bacterial proteins (Rabiet et al., 2005). Under normal conditions, all mitochondrial components are located inside mitochondria. However, during stress or damage, some of them are released in cytosol and recognized by pattern recognition receptors (PRRs) due to the bacterial origin. That is why mitochondria are considered a powerful regulator of the inflammatory response (Meyer et al., 2018).

It was established in the hippocampus of the rat PTSD model, that lysosome function was enhanced which may serve as a kind of protection mechanism against the accumulation of damaged mitochondria (Wan et al., 2016). Programmed elimination of damaged mitochondria in living cells has been called “mitoptosis” (Mijaljica et al., 2010). It is believed that there are two main ways of mitoptosis: destruction of the OMM or the IMM. This process can be associated with both apoptosis and autophagy. Damaged mitochondria can often be digested by lysosomes to aid cell survival but if autophagic degeneration is also takes place, it can result in cell death (Tinari et al., 2007). If the cell survives, mitochondrial biogenesis, a self-renewal pathway by which new mitochondria are generated from existing ones, can potentially be activated. Number of mtDNA copies in cell is a marker of mitochondrial biogenesis (Popov, 2020; Preston et al., 2020). There are no virtualy data on number of mtDNA copies in the brain under PTSD conditions. One study found that pregnant women with symptoms of PTSD (prenatal PTSD) had a reduced number of mtDNA copies in the placenta. They associated the decreased number of mtDNA copies with the possible development of oxidative stress after a traumatic event. Such features of pregnant women with PTSD can potentially affect the future generation (Brunst et al., 2017). In veterans with PTSD significantly lower mtDNA copy numbers in granulocytes was found (Bersani et al., 2016). Veterans with moderate symptoms had higher levels of mtDNA compared to PTSD veterans with mild and severe symptoms, but no difference with controls. This may be due to the fact that under conditions of mild cellular stress, mitochondrial biogenesis is activated as a compensatory mechanism, whereas under conditions of severe stress, it leads to damage and dysfunctions of mitochondria (Bersani et al., 2016).

**FIGURE 3:**
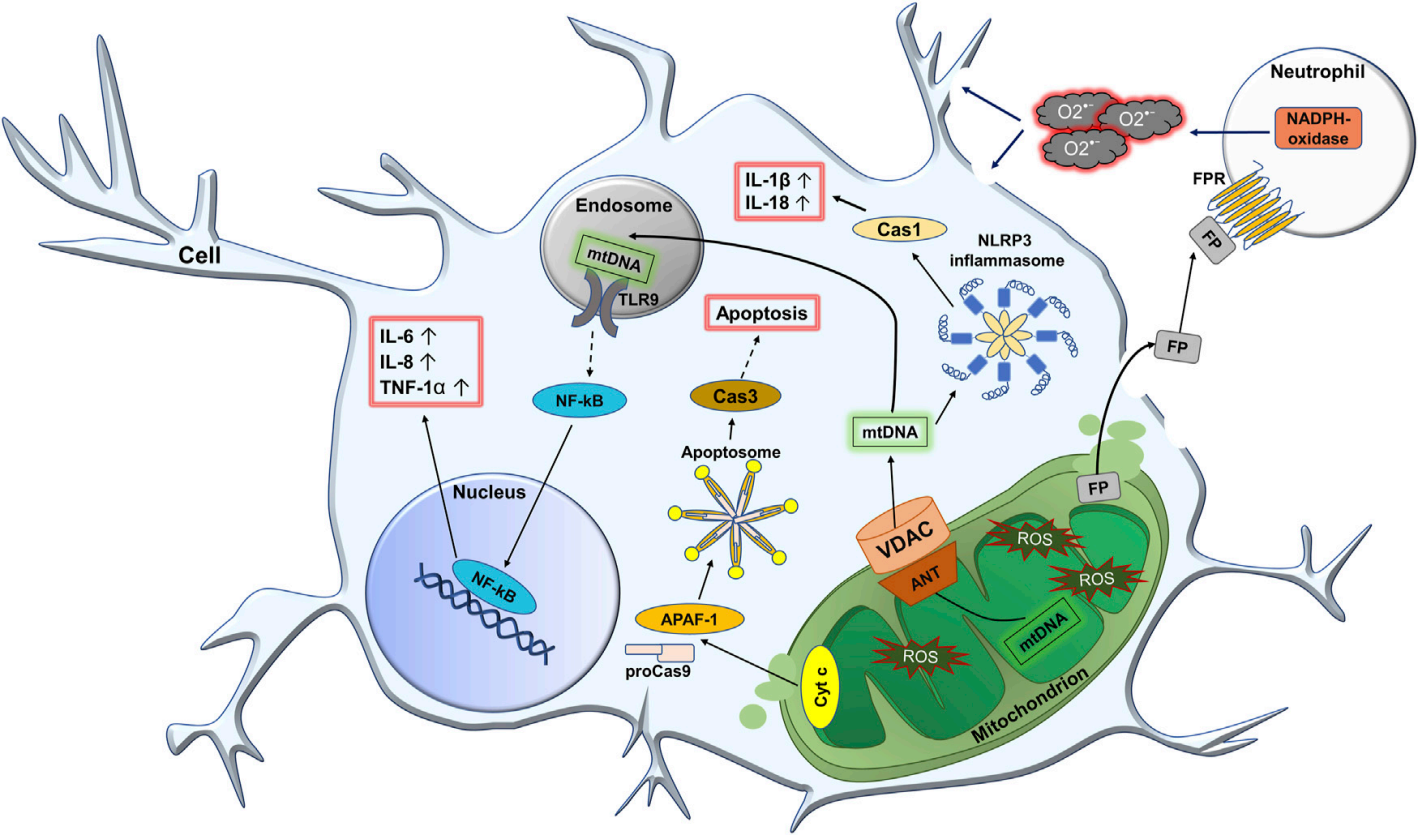
Potential role of mitochondria in the development of neuroinflammation at PTSD. In PTSD, the production of reactive oxygen species (ROS) in nerve cells increases. First of all, the number of oxidatively modified biomolecules inside the mitochondria increases. This leads to the formation of harmful mitochondrial DAMPs/PAMPs (damage/pathogen associated molecular patterns), such as oxidized mitochondrial DNA (mtDNA), N-formyl peptides (FP), cytochrome *c* (Cyt *c*), etc. Due to the increased permeability of the mitochondrial membrane, DAMPs/PAMPs can freely pass through the damaged mitochondrial membranes and, in particular, through the pore consisting ANT (adenine nucleotide translocase) protein on the inner membrane and VDAC (voltage dependent anion channel) on the outer membrane. Once in the cytoplasm, mtDNA can activate the NLRP3 (NLR family pyrin domain containing 3) inflammasome, which leads to the activation of Cas 1 (caspase 1). In turn, this increases the level of active forms of IL-1β and IL-18. Also, upon entering the endosome, mtDNA is recognized by TLR9 (toll-like receptor 9), which leads to the activation of the transcription factor NF-kB (nuclear factor kappa light chain enhancer of activated B cells). The latter is further translocated into the nucleus where it activates the expression of pro-inflammatory cytokines IL-6, IL-8, and TNF-1a. When Cyt c is released into the cytoplasm, together with Apaf-1 (apoptotic protease-activating factor-1) and pro-caspase-9 (proCas9), it participates in the formation of the apoptosome. At the same time, proCas9 is activated and further activates caspase 3 (Cas3), which leads to the development of apoptosis. In addition, when the inflammatory process leads to severe cell damage, mitochondrial FP can be released externally. FP interacts with the N-formyl peptide receptor (FPR) on the surface of leukocytes, especially neutrophils resulting in their activation. This activation promotes NADPH-oxidase of neutrophils to increase production of superoxide anion radical O_2_^•−^, that attacks the cell from the outside.

In general, mitochondrial dysfunctions lead to production and release of mtDAMPs/PAMPs, leading to development of neuroinflammation which will be covered with details below. At the same time, the activation of lysosomal function can serve as a kind of protective mechanism against accumulation of damaged mitochondria. Figure 3 demonstrates potential role of mitochondria in the development of neuroinflammation at PTSD.

##### 1.2.2.2 Activation of TLR9/NF-kB pathway

It is known that mtDNA can activate TLR9/NF-kB pathway because it is directly recognized by Toll-like receptor 9 (TLR9). These receptors are classified as intracellular due to their localization on endosomes. They are mainly expressed in antigen-presenting immune cells but are also found in most cells of the body. Nucleic acids are ligands for TLR9. That is, among mtDAMPs/PAMPs the only mtDNA, that entered the endosome from the cytoplasm and can be recognized by TLR9 (Karapetyan et al., 2020). Such interaction between mtDNA and TLR9 triggers a cascade of reactions that ultimately leads to the activation of nuclear factor-kappa B (NF-kB) (Kawai and Akira, 2007). NF-kB is a transcription factor that regulates the expression of genes involved in the cellular inflammatory response. Its activity is strictly regulated. The main mechanism of its regulation is associated with the inhibitory protein called IkB (inhibitor of kB). Under normal conditions, NF-kB is located in cytosol in the form of an inactive NF-kB/IkB complex. Degradation of the IkB inhibitor is a crucial point for nuclear translocation of NF-kB. NF-kB contains a so-called nuclear localization signal (NLS), and IkB contains a nuclear export signal (NES), due to which the NF-kB/IkB complex is constantly exported from the nucleus to the cytosol (Oeckinghaus and Ghosh, 2009). Recognition of mtDNA by TLR9 quickly leads to activation of the IkB kinase (IKK) multiprotein complex, that phosphorylates the inhibitory protein IkB. Phosphorylated IkB is then ubiquitinated and degraded by proteasoma (Kawai and Akira, 2007). After degradation of IkB, free NF-kB due to the presence of NLS in its structure is translocated into nucleus, remains there and operates as a transcription factor activating expression of genes encoding pro-inflammatory cytokines such as IL-6, IL-8, and TNF-1α. It induces the inflammatory process (Oeckinghaus and Ghosh, 2009).

The function of NF-kB in the brain has currently not received sufficient attention. However, it has been reported that NF-kB is activated in chronic neurological disorders (Shih et al., 2015) and long-term activation of NF-kB in the hippocampus caused PTSD-like behavior that was reduced by an NF-kB inhibitor (Gupta and Guleria, 2022). PTSD was found to be related inability of TLR9 to evade the pro-inflammatory NF-kB pathway (Zimmerman et al., 2012). However, in addition to the NF-kB pro-inflammatory pathway, there is a so-called alternative homeostatic pathway that suppresses production of pro-inflammatory cytokines (Zimmerman et al., 2012). This indicates the possibility for pleiotropic effects.

##### 1.2.2.3 NLRP3 inflammasome triggering

mtDNA can activate the NLRP3 inflammasome (nucleotide-binding oligomerization domain (NOD), leucine-rich repeat (LRR)-containing proteins 3 (NLR)). The latter is a cytosolic protein immune complex that is part of innate immunity system. The main components of NLRP3 inflammasome are sensor (NOD-like-receptors (NLRs)), which recognize ligands, adapter protein (apoptosis-associated speck-like protein containing a CARD (ASC)), and procaspase 1, which is an effector (Hamarsheh and Zeiser, 2020). Inflammasomes are formed in two stages: priming and activation. The priming stage is characterized by transcriptional activation of zymogens (inactive precursors) of pro-inflammatory cytokines pro-IL-1β and pro-IL-18, as well as upregulation of NLRP3 transcription. The latter is activated by NF-kB (McKee and Coll, 2020). Activation of the immune complex with the participation of mitochondria begins with the recognition of mtDNA, that is a ligand for NLRs (Zhang et al., 2022). This leads to activation of the central nucleotide-binding domain and its oligomerization. After that, the central domain recruits the ASC adapter protein. ASC contains a caspase recruitment domain (CARD) and binds to pro-caspase-1, which also contains a CARD domain (CARD-CARD interaction). That induces autoproteolysis of pro-caspase-1, which is converted to its active form—caspase-1 (Blevins et al., 2022). Caspase-1 is a cysteine protease that ensures the maturation of proinflammatory cytokines. In particular, it converts pro-IL-1β and pro-IL-18 into their active forms IL-1β and IL-18, that initiate an inflammatory response (Molla et al., 2020).

There is an increasing body of evidence that activation of the NLRP3 inflammasome plays a crucial role in the pathophysiology of PTSD and the development of brain inflammation. For example, higher expression of NLRP3 and caspase-1 was observed in astrocytes of mice with PTSD-like behavior (Ji et al., 2022). This suggests that the NLRP3 inflammasome pathway was indeed activated in nervous tissue under PTSD conditions with a concomitant increase in ROS levels in mitochondria. In rodents, at PTSD the level of hippocampal IL-1β was elevated, and the use of immunomodulatory preparations reduced it along with a decrease anxious behavior that is related to this disorder (Waheed et al., 2018). No relationship between circulating blood levels of IL-18 and PTSD was found, whereas IL-1β was associated with PTSD and its level was higher (Baumert et al., 2013).

##### 1.2.2.4 Induction of apoptosis

Activation of apoptosis is another possible mechanism relating stress and PTSD. Numerous oxidative modifications to mitochondria can lead to their dysfunction and release of cytochrome *c*. Cytochrome *c* is a heme-containing protein that is located normally on the IMM and its main function is the transport of electrons between complex III (cytochrome bc1) and complex IV (cytochrome *c* oxidase) of the electron transport chain. It is loosely connected to the IMM by electrostatic bonds, and therefore, in case of mitochondrial damage, it may detach and enter the cytoplasm (Garrido et al., 2006). In the cytosol, there is the protein called apoptotic protease-activating factor-1 (Apaf-1) that is a particular sensor of cytochrome *c* in the cytoplasm. In the presence of ATP or dATP, Apaf-1 and cytochrome *c* assemble into a large apoptosome complex. The main function of the apoptosome is regulation of the catalytic activity of caspase-9 (Cas9). In the cell, Cas9 is normally kept in the form of a zymogen—pro-caspase-9. Apaf-1 contains the above-mentioned CARD in its structure. Due to this, the apoptosome interacts with the pro-caspase-9 and triggers autocatalytic activation to Cas9 (Bao and Shi, 2007). Caspases are a family of cysteine proteases that mediate programmed cell death. Cas9 belongs to the initiator caspases, that activate other effector proteases, in particular, caspase-3 (Avrutsky and Troy, 2021). Inactive caspase 3 is a dimer and processing of the interdomain linker under the action of Cas9 leads to its conformational change and activation. After that, Cas3 can cleave key structural proteins, cell cycle proteins, and DNases, that leads to DNA fragmentation, formation of apoptotic bodies, and cell death (Ponder and Boise, 2019).

To date, we have been able to find at least four researches on the relationship between cytochrome *c* and PTSD models (Li et al., 2010; Xiao et al., 2011; Liu et al., 2012; Garabadu et al., 2015). It has been established that a higher expression of cytosolic cytochrome *c* was observed in the brain of rats with PTSD (Li et al., 2010; Liu et al., 2012). At the same time, enlarged, swollen, fragmented cristae, damaged outer membrane and signs of vacuolar degradation of mitochondria were clearly visible along with higher expression of caspases 3 and 9 (Li et al., 2010; Xiao et al., 2011). This suggests that apoptosis occurs in PTSD. However, unlike necrosis, apoptosis is programmed cell death, preventing the development of inflammation. Despite this, it may be impossible to avoid the development of inflammation if activation of immune cells takes place.

##### 1.2.2.5 Activation of immune cells

Mitochondrial DAMPs/PAMPs can activate immune cells. Firstly, this is due to the fact that mitochondria have a bacterial origin and retain pro-inflammatory bacterial molecular motifs. Among them, the so-called N-formyl peptides (FP), possessed by bacteria, are important for the activation of leukocytes. FP are formed as a result of cleavage of mitochondrial proteins. When a cell is damaged, they are released from mitochondria and can activate an inflammatory response through interaction with the N-formyl peptide receptor (FRP) (Lubkin et al., 2018). In general, these FPs are powerful chemoattractants for immune cells, in particular, for phagocytes, such as monocytes and neutrophils, directing them to the sites of inflammation (Campbell et al., 2018). FRPs are prominently expressed in nervous tissue, particularly in the central nervous system (Cussell et al., 2020). They belong to transmembrane G protein-coupled receptors, that bind peptides with N-formylated methionine, representatives of bacterial or mitochondrial proteins (Chen et al., 2022). As a result of the interaction of the ligand with FRP, that is expressed on phagocytes, activation of effector cells, such as neutrophils, occurs (Cao et al., 2023). Normally, neutrophils are at rest, and their activation is the driver of the inflammatory process. In response to an interaction of the chemoattractant with FRP, numerous intracellular signaling pathways are activated, leading to migration of neutrophils to the sites of inflammation, where phagocytosis and ROS formation take place (Mayadas et al., 2014). Phagocytosis maintains homeostasis due to a rapid clearance of cellular debris and apoptotic bodies (Westman et al., 2020). In parallel with phagocytosis, the NADPH oxidase enzyme is activated. This is a multicomponent enzyme that acquires catalytic ability only after assembly of its cytosolic components with transmembrane components. This leads to the generation of significant amounts of the superoxide radical. Excessive activity of NADPH-oxidase can cause a long-term inflammatory response (Belambri et al., 2018).

In general, it is currently believed that the nature of chronic neuroinflammatory conditions is closely related to the activation of FRPs. In particular, this is associated with neurodegenerative diseases such as Alzheimer’s or Parkinson’s diseases (Cussell et al., 2020). This has not yet been established for PTSD although, for example, it is known that circulating levels of neutrophils and monocytes in the blood of PTSD patients are significantly higher compared to controls. Changes in the levels of lymphocytes, as well as other granulocytes, such as eosinophils and basophils, were not observed (Lindqvist et al., 2017). Potentially, this may indicate activation of immune cells mediated by FPR. In addition, in the rat PTSD model, higher expression of NADPH oxidase subunits was observed in the amygdala. This was accompanied by the development of oxidative stress, evidenced by, lower SOD activity, decrease in the concentration of reduced glutathione and increase in the level of malondialdehyde compared to the control group (Petrovic et al., 2018).

### 1.3 Mitochondria as a potential target for the treatment of PTSD-related inflammation

Characteristic symptoms of PTSD are an exaggerated sense of fear and re-experiencing a stressful event through intrusive thoughts and nightmares. This leads to the occurrence of chronic stress, which cannot be avoided on your own. It not only affects hormone levels but also changes the levels of inflammatory markers and in general, such changes in the organism can lead to PTSD development (Maeng and Milad, 2017).

As we described above, mitochondria may be significantly involved in the development of inflammation among PTSD patients. This raises the question: could mitochondria be a potential target for the prevention and treatment of inflammation? In fact, it is quite difficult to find a drug that would act specifically on mitochondria. However, there is the study linking PTSD treatment to inflammation and mitochondria. In 2022, scientists proposed leptin as a candidate for the pharmacological treatment of PTSD (Ji et al., 2022). First, this was due to the disclosed fact that administration of leptin to mice with PTSD-like symptoms, decreased the level of ROS, increased the level of ATP in astrocytic mitochondria, and inhibited the activation of astrocytic NLRP3 inflammasomes. Second, leptin was associated with effective improvements in behavioral responses, including fear memory, cognitive impairment, and depressive-like behavior (Ji et al., 2022). In another study (Garabadu et al., 2015), scientists evaluated the drugs called Risperidone and Paroxetine, to assess their effects on mitochondrial dysfunction and mitochondrial-dependent apoptosis in specific brain regions in a modified rat model of PTSD. Risperidone in a medium dose ameliorated an increased in activity of the mitochondrial electron transport chain, and decreased mitochondrial membrane potential, levels of cytochrome *с*, caspase-9, and 3 in the hippocampus, hypothalamus, prefrontal cortex and amygdala. Paroxetine, unlike risperidone, did not affect mitochondrial function but reduced the intensity of mitochondrial oxidative stress. However, due to the effective prevention of mitochondrial dysfunctions, both preparations showed an anti-apoptotic effect, that was accompanied by a decrease in depressive symptoms, anxiety and cognitive deficits (Garabadu et al., 2015). On the other hand, there is a non-pharmacological way of treating of mitochondrial dysfunction in PTSD. In particular, in 2019, the effectiveness of physical exercise was established. For their study, scientists used a rat model of PTSD (Seo et al., 2019). Exposure to severe stress induced mitochondrial dysfunction in the hippocampus, including disruption of Ca^2+^ homeostasis, increased H_2_O_2_ level, decreased O_2_ respiration rate, and overexpression of mitochondrial pore proteins. Physical exercise improved all of these markers, and also contributed to the reduction of anxiety, depression, and cognitive impairment (Seo et al., 2019).

Overall, the relationship between mitochondria, inflammation, and PTSD is largely unexplored. However, mitochondria damaged by trauma or oxidative stress may be involved in the development of inflammation in PTSD, and treatment of mitochondrial dysfunction correlates with effective treatment of PTSD-like symptoms. That is why we believe that mitochondrial dysfunctions associated with PTSD need more attention, and methods of their prevention should be used in the complex therapy of PTSD and propagated between affected populations.

## 2 Conclusion and perspectives

Development of mitochondrial dysfunctions is one of the key components of mental disorders, including PTSD. In 2015, German scientists found that some single nucleotide polymorphisms (SNPs) in mitochondrial genes may be associated with its occurrence (Flaquer et al., 2015). They identified two SNPs that were found in people with PTSD: mt12501 C→T located in the NADH dehydrogenase subunits 5 (MT-ND5) and mt8414 C→T located in adenosine triphosphate (ATP) synthase subunit 8 (MT-ATP8). These results highlight the important role of the mitochondrial genome as a risk factor for the development of PTSD. Potentially, the presence of these mtSNPs may increase susceptibility to this disorder (Flaquer et al., 2015). Both proteins are associated with oxidative phosphorylation and generation of ROS. NADH dehydrogenase is generally considered as the main site of ROS production in the mitochondria (Zhao et al., 2019). Actually, the oxidative stress observed in PTSD may underlie its pathophysiology (Lushchak et al., 2023; Reed and Case, 2023).

Numerous forms of oxidative damages to mitochondria can lead to an increase in permeability of their membranes and release of mtDAMPs/PAMPs into the cytoplasm or, under certain conditions, outside the cell. It is believed that due to their bacterial origin, they are recognized by PRRs, which include NLRs, TLRs, and FPRs. In turn, this leads to activation of the main pathways of inflammation: activation of NLRP3 inflammasome, TLR9/NF-kB pathway, and activation of neutrophils, respectively. Their study in PTSD undoubtedly needs more attention, since the number of studies linking them is insufficient to understand the mechanisms of induction and further development of neuroinflammation. In addition, the release of cytochrome *c* from the mitochondria into the cytoplasm provokes apoptosis. Specifically, this mechanism has already been confirmed in PTSD, which explains the large number of apoptotic cells in the brains of these patients.

In addition to the fact that mitochondria directly regulate the immune response of the cell, they can potentially do this through participation in the biosynthesis and catabolism of hormones involved in the development of PTSD. First of all, this disorder is associated with an imbalance in the work of the HPA axis and, in particular, changes in the level of cortisol and epinephrine. Changes in the levels of other catecholamines such as norepinephrine and serotonin, are also often mentioned. *To date, no reliable specific biochemical PTSD markers have been identified*, since this disease is characterized by a huge range of symptoms and indicators, that can be completely opposite in different patients. This greatly complicates the search of markers and causes of PTSD pathophysiology.

We hypothesize that changes in mitochondrial protein activity/expression may influence hormone levels in PTSD. Our hypothesis is that due to the lower expression of TSPO, the precursor of steroid hormones is poorly transported into the mitochondria, and the lower expression of CYP450 limits the first step of steroidogenesis, which may lead to lower cortisol levels. A significant limitation of this assumption is the lack of data on the determination of these parameters in the adrenal glands. Since PTSD is a neuropsychiatric disease, the mentioned parameters need to be determined in the brain, however, steroidogenesis occurs in the adrenal glands. This is why we believe that researchers should also pay attention to the connection between the adrenal glands and PTSD. Hence, some blood, stool, and urine components could potentially be used for characterization of PTSD.

It is noteworthy that the mitochondrial enzyme MAO A provides oxidative deamination of epinephrine, norepinephrine, and serotonin. In PTSD, MAO A activity can also be changed and, in particular, it is known to be lower compared to healthy people. In turn, this can affect the concentration of the above-mentioned hormones. Since cortisol and norepinephrine are considered as anti-inflammatory hormones, and serotonin is considered pro-inflammatory, changes in their relative levels could cause/influence inflammation. Moreover, their ratio may serve as a potential PTSD marker.

We believe that scientists underestimate the role of mitochondria in the development of PTSD. Given a role for mitochondrial dysfunctions in the pathophysiology or PTSD, potentially treating patients with a combination of standard therapy and treatments for mitochondrial dysfunctions could prevent or alleviate chronic inflammation. This systematic review may provide new insights for future research and contribute to a broader study of the relationships between mitochondria, inflammation, and PTSD, as well as new approaches to PTSD treatment.
